# Head-eye movement of collegiate baseball batters during fastball hitting

**DOI:** 10.1371/journal.pone.0200443

**Published:** 2018-07-17

**Authors:** Takatoshi Higuchi, Tomoyuki Nagami, Hiroki Nakata, Kazuyuki Kanosue

**Affiliations:** 1 Fukuoka Institute of Technology, Faculty of Socio-Environmental Studies, Fukuoka, Japan; 2 Kitasato University, College of Liberal Arts and Sciences, Sagamihara, Japan; 3 Nara Women’s University, Faculty of Human Life and Environment, Nara, Japan; 4 Waseda University, Faculty of Sport Sciences, Tokorozawa, Japan; The Australian National University, AUSTRALIA

## Abstract

Successful baseball hitting involves a combination of highly trained perceptual skills and forceful bat swing motions. The purpose of the present study was to quantify the horizontal movement of the head and eyes while baseball batters hit a fastball to clarify a visual strategy for this highly trained interceptive task. Six collegiate baseball players hit a fastball that was launched from a pitching machine. The ball speed was 31.9 m·s^-1^ for the Slow Ball Task and 40.3 m·s^-1^ for the Fast Ball Task. Horizontal head movements were analysed using images that were captured by two high-speed video cameras. The Horizontal eye movement was recorded with electrooculography. The angular speed of the horizontal head and eye movements during hitting were divided into four time periods (I-40 = 21–40% of total ball-flight, I-60 = 41–60% of total ball-flight, I-80 = 61–80% of total ball-flight, I-100 = 81–100% of total ball-flight) and analysed using analysis of variance and a Tukey post-hoc multiple-comparison. In the Slow Ball Task, the horizontal angular velocity of the head during I-80 was significantly faster than that during I-40 (p < 0.05). In the Fast Ball Task, the horizontal angular velocity of the head during I-80 was significantly faster than that during I-40 and I-60 (p < 0.05). These results indicated that the tracking motion of the head became faster as the launched ball came close to the batters, but there was no change in the angular tracking motion of the eyes. Therefore, rapid eye movement may not be suitable to accurately estimate the ball’s future location during fastball hitting based on the eye-centered coordinates. Our findings suggest that conventional vision training with a wide range of saccadic or smooth-pursuit eye movements does not reflect the characteristics of tracking strategies during baseball hitting.

## Introduction

Intercepting a rapidly moving object with a tool requires accuracy both for estimating the object’s trajectory based on visual information and manipulating the tool at a fraction of the time. The key components that are required for successfully intercepting a rapidly moving object are experience in seeing the object’s flight, an internalized estimate of the Earth’s gravitational force, and actual visual information about the object during the interceptive task [1–4]. In some sports such as baseball, tennis, and cricket, players must match the ball’s trajectory and bat (racket) swing with temporal and spatial accuracy. Hitting a ball with a round bat in baseball is considered one of the most difficult skills in sports [5] because professional hitters, even those with a success rate (batting average) of 0.300, can be categorized as “elite players.” Therefore, investigating an experienced batter’s ability to hit a ball accurately will provide with knowledge about operating at the utmost limit of human performance in a difficult task. Moreover, clarifying the batter’s strategy about seeing the ball will suggest specific perceptual and/or motor components that might contribute to our understandings of a baseball batter’s hitting performance.

Years of hitting experience make it possible for batters to accurately estimate when and where to swing a bat based on pitch trajectory can be seen before the initiation of bat swing [6, 7]. In addition, several studies investigated visual strategies for batters to keep their eyes on the pitcher and pitched ball. Bahill and LaRitz reported that baseball batters used smooth-pursuit eye movement and head movement to track a simulated fastball without bat swing [8]. In the non-sports laboratory setting, intercepting a moving target or predicting a visual motion with accuracy is better when the smooth-pursuit eye movement was utilized [9, 10]. In cricket, the superior abilities to couple the direction of the head to the moving ball and the predictive saccadic eye movement to the ball bounce and ball-bat contact location were reported as characteristics of elite cricket batters [11, 12]. Research on experienced baseball batters who watched a pitching video found that the expert batters’ eyes were fixated on the ball’s anticipated release point during wind-up, and then they moved their fixation point to approximately 150 ms after the ball was released [13, 14]. Uchida et al. examined the dynamic visual acuity of baseball players compared with that of non-baseball players and found that the speed of their horizontal eye movements, rather than the perception of moving images on the retina, contributed for superior dynamic visual acuity [15]. These studies showed that successful baseball hitting involves a combination of highly trained perceptual skills and forceful bat swing motions. However, these previous studies focused on these factors separately. How an experienced batter’s perceptual skills interact with their well-trained bat swing motions when they attempt to hit a ball is poorly understood. A further investigation is necessary because previous reports were based on batters’ head-eye motions when they did not swing a bat. When a batter tries to hit a ball, the torso rotates horizontally in the opposite direction of the pitch. Motions that are involved in the swing possibly affect the head-eye movements that are used to track the ball. Although the actual tracking strategies of experienced baseball batters are still unknown, the rapidity of the smooth-pursuit component of eye movements should be necessary for successful interception [9, 10]. Some conventional vision trainings that aim to improve the range and speed of eye movements without any coordinative action of other body parts have been introduced to baseball batters. Clarifying the batter’s strategy of seeing the ball while they are hitting will indicate the abilities that are necessary for accurately intercepting the ball.

The purpose of the present study was to quantify the horizontal movement of the head and eyes while baseball batters hit fastballs to determine a visual strategy for this highly trained interceptive task. The horizontal component of the head-eye movement was selected because a thrown ball mainly travels in horizontal direction from a batter’s perspective. Difference between the head and eyes direction and a calculated ball direction from the batter’s perspective was utilized to quantify the batter’s tracking performance. Speeds of the head and eyes horizontal movement in four periods before hitting a ball were compared in order to clarify the contribution of each component for tracking. Since wide range of pitched ball speed exists in actual competition of baseball, two tasks, one with a relatively slow ball speed and another with a relatively fast ball speed in the actual baseball game, were selected.

## Materials and methods

The experimental protocol and risks were explained to all of the participants before their involvement in this study. Written consent was obtained from all participants. The participants were naive to the purpose and benefits of the experiment until a post-experiment debriefing was given. All experimental procedures received approval from the Ethics Review Committee on Human Research of Waseda University (approval number: 2010043).

### Participants

Six collegiate baseball field players (three right-handed batters and three left-handed batters) participated in the experiment. The mean age, height, and body mass (mean ± standard deviation [SD]) were 20.7 ± 1.5 years, 1.79 ± 0.05 m, and 78.0 ± 5.7 kg, respectively. Their length of baseball experience (mean ± SD) was 11.3 ± 1.4 years (range, 9–13). The participants performed the experiment in one day and were instructed to eat and drink water normally one day before and on the day of the experiment. In addition, they were told to sleep normally the night before the experiment, and each participant was asked about their physical and mental states before the experiment. All of the participants responded that their physical and mental states were good or normal.

### Materials

**Fig 1:**
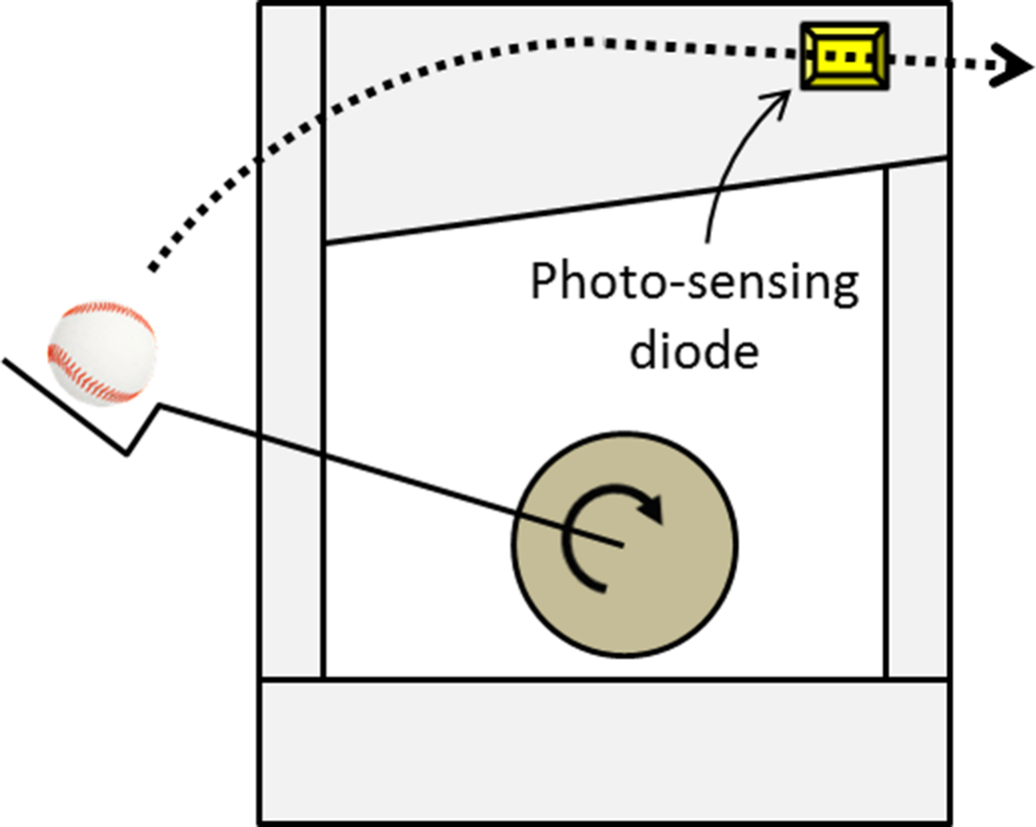
The arm-style pitching machine with a photo-sensing diode.

An arm-style pitching machine (NA61, Nisshin SPM Ltd., Japan) launched a ball from 17 m (55.77 feet) away from the home plate and 1.6 m (5.25 feet) above the ground. The pitching machine was set to launch a ball with backspin, to replicate the typical fastball trajectory. The participants were given the choices of four types of wooden bats (length = 840 mm; 33.01 inches, mass = 900 g; 31.75 ounces) with different shapes. A photo-sensing diode (PLDM-10, Sankei Machinery, Japan, Fig 1) was mounted on the pitching machine to detect and record the moment the ball was released. To detect the moment at which the ball and bat made contact, we placed a sound-to-electrical transducer trigger unit (ATRG-100, Nihon Fastec Imaging co., Ltd, Japan) behind the home plate.

### Tasks

After sufficient warm-up and practice hitting, the participants hit fastballs ten times at two different ball speeds. The two ball speeds, which were considered relatively fast and slow ball speeds during actual competition, were adopted to investigate the participant’s hitting performance under two tasks. In the slow speed task, ten trials with a launched ball speed of 31.9 m·s^-1^ (115 km·h^-1^; 71.8 MPH) were performed (Slow Ball Task). For the fast speed task, ten trials with a launched ball speed of 40.3 m·s^-1^ (145 km·h^-1^; 90.7 MPH) were performed (Fast Ball Task). The order of presentation of the two blocks was randomized among participants, wherein they hit either slow balls or fast balls ten times in a row per ball speed, with the type of ball speed randomly determined. The speeds were measured with a radar gun (The Jugs Company Japan Ltd., Japan) for adjustment. The order of the two tasks was randomized. The participants were instructed to place their trailing (back) foot at their preferred position and keep the foot in the same position during all of the trials.

### Electrooculography

Horizontal eye movements were determined using electrooculogram (EOG) signals. Two Ag-Ag Cl surface electrodes were positioned on the participant’s temples on both sides of the head to detect changes in polarity during the horizontal eye movement. A ground electrode was positioned on the spinous process of seventh cervical vertebra. The EOG signals were recorded at 1000 Hz, amplified (MEB-2216, Nihonkohden, Japan), and filtered with a low pass filter with a cut-off frequency of 50 Hz. The 50 Hz low-pass filter was selected to remove electrical noise and artifacts without affecting the essential frequency of the eyes’ electrical activity [16]. The signals were then transferred to a data acquisition system (PowerLab, AD Instruments Japan, Japan) and recorded using a data analysis software program (Chart 5.5, AD Instruments Japan, Japan) on a personal computer. Signals indicating the ball release and ball-bat contact were also recorded. Before recording data, to test the accuracy and reliability of this measurement method, we analysed the EOG activities while one experimenter made horizontal saccadic eye movements with 15°, 30°, 45°, and 60° five times each. The linear relationship between the average EOG activity in each setting and angular displacement of the eyes was confirmed (r = 0.99). To ensure the consistency of the EOG data, during every trial, the participants were asked to look at a sign on the pitching machine for approximately 2 seconds before the ball was released.

### Recording the ball, bat, and head motions

The movements of the ball and two markers on the bat were recorded during hitting with two high-speed video cameras (frame rate = 1000 Hz, exposure time = 0.5 ms, resolution = 680 × 480 pixels, Trouble Shooter, Fastec Imaging Corporation, USA) which were electrically synchronized. One camera was placed 6 m away from the home plate at a right angle to a line between the center of the pitching rubber and the center of the home plate, and the other camera was placed 6 m behind the home plate to provide a rear view of the hitting movement. The hitting motion was recorded for 150 ms before and 150 ms after a sound-to-electrical transducer trigger unit registered the ball-bat contact. The markers were attached to the barrel end of the bat and 450 mm down from the barrel end of the bat to aid in the analysis of the data. Hitting motions were recorded for 150 ms before and 150 ms after the ball made contact with the bat. Using the protocol of a previous study [7], we placed cameras at suitable locations to track and analyse the ball and bat motions. To record the participant’s head movements, we attached three markers to their batting helmet. One of the three markers was placed at the topmost point of the helmet. Another marker was placed at the most anterior point of the visor. The third marker was placed on the lateral edge of the visor where the visor joined the helmet. This marker was located on the left side for left handed batters and the right side for right handed batters. The positions of the markers were captured with two high-speed video cameras (frame rate = 300 Hz, resolution = 640 × 480 pixels, GC-PX1, JVC, Japan).

### Data analysis

**Fig 2:**
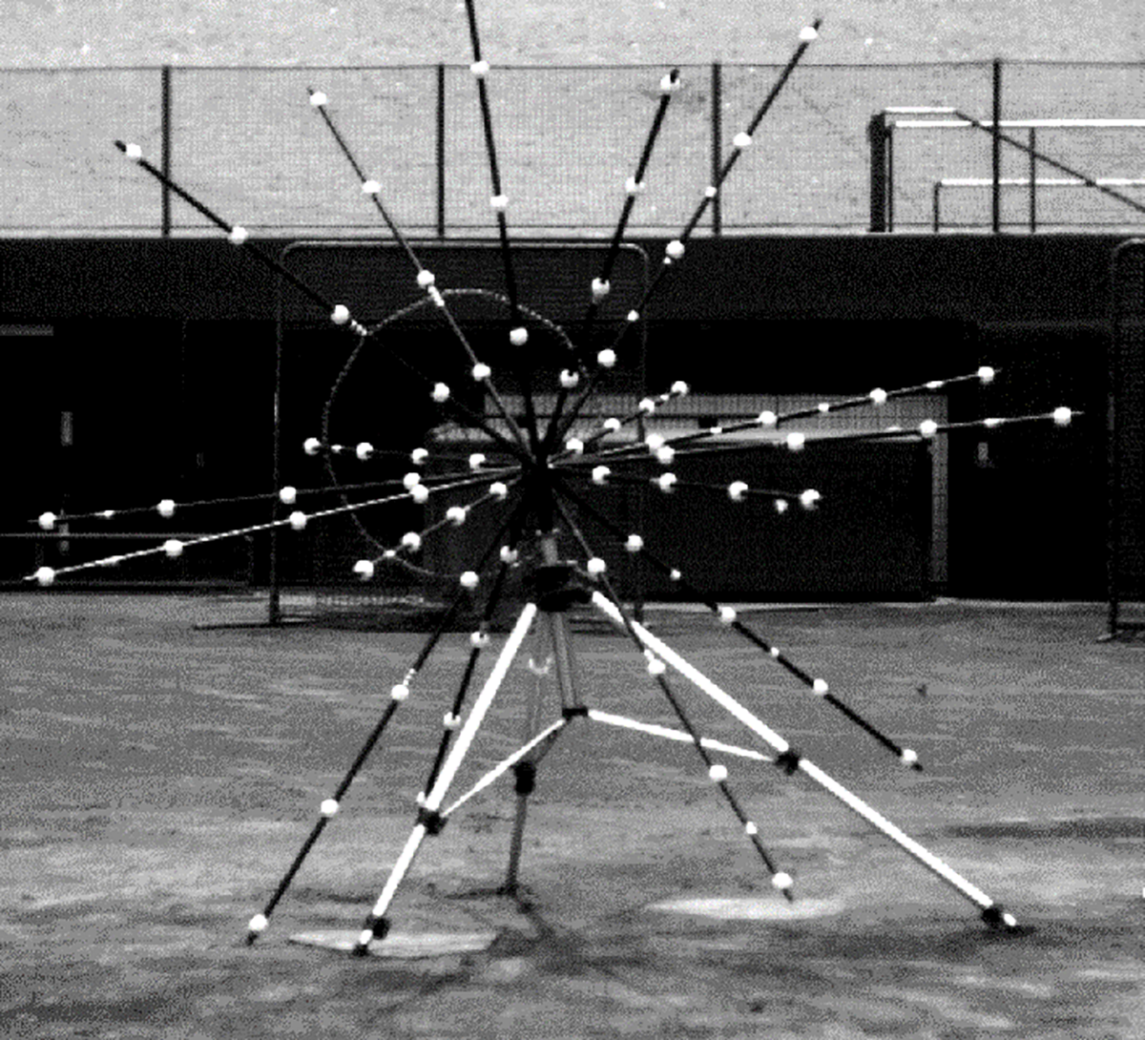
A radial calibration structure.

**Fig 3:**
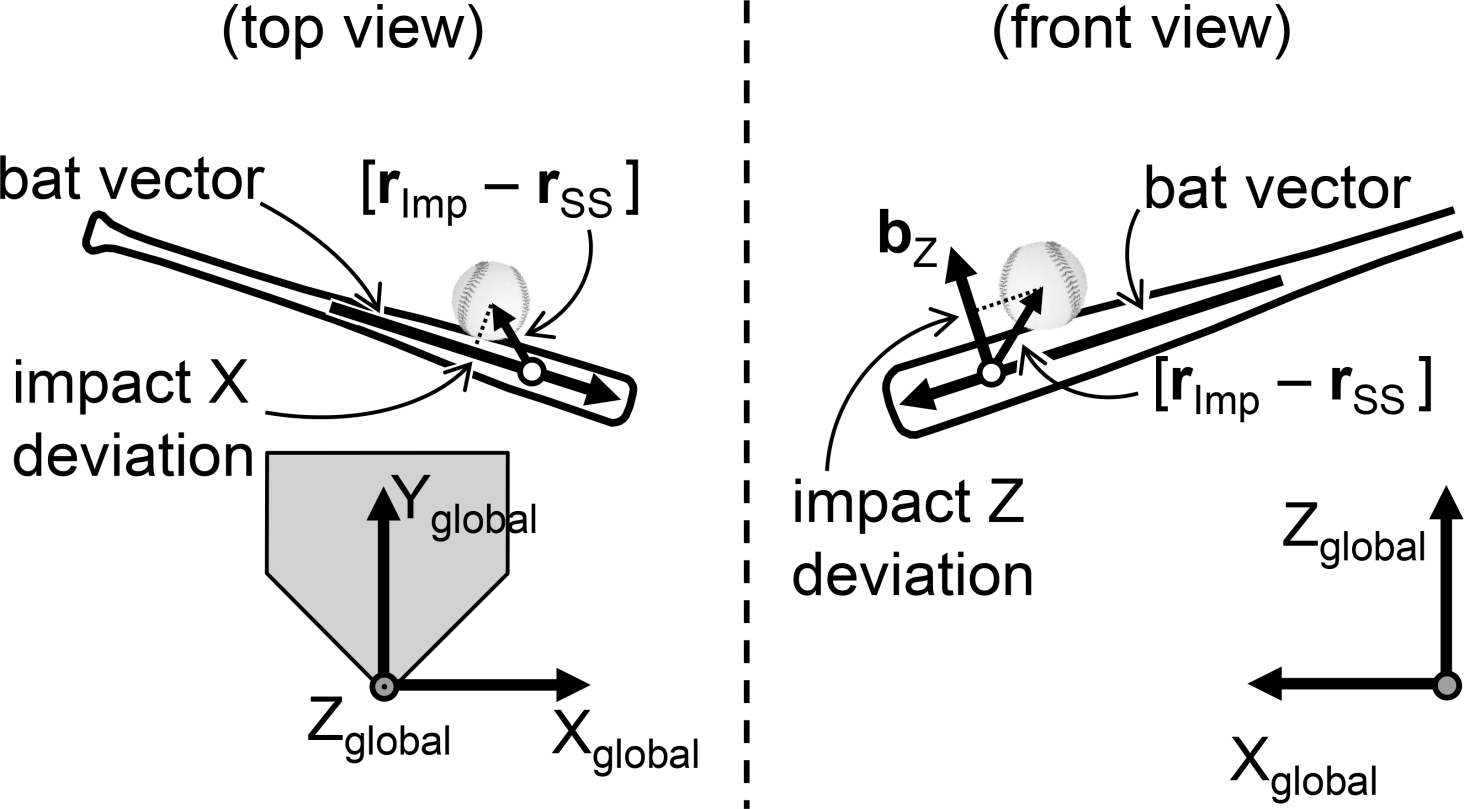
Definition of the global coordinate system and the bat coordinate system. The drawing represents the instant of ball-bat contact from the top view (left) and front view (right). Impact X deviation is the distance between the sweet spot of the bat (white circle; r_ss_) and the center of the ball (r_Imp_) in the direction of bat vector. Impact Z deviation is the distance between the sweet spot of the bat (white circle; r_ss_) and the center of the ball (r_Imp_) in the direction of b_z_.

Data about the location of the launched ball and markers on the barrel and grip end of the bat, and helmet were obtained from the high-speed video camera images and analysed using a motion analysis system, Frame Dias IV (DKH, Japan). Three-dimensional coordinates were obtained using the direct linear transformation method with 5 reference markers that consisted of vertical markers set at 0.5 m, 1.0 m, and 1.5 m above the rear point of the home plate, and the horizontal markers were set at 0.5 m and 1.0 m from the rear point of the home plate to pitcher’s plate with height of 0.5 m. A radial calibration structure that was approximately 2 m × 2 m ×2 m in size, with 68 reference points (Fig 2) was used to improve the spatial accuracy of the measurement. To determine the spatial relationship between the bat’s sweet spot and ball at the point of impact, the methodology that was described in a previous study was used to establish the global and bat coordinate systems [7]. The right-hand orthogonal reference frame was defined by the X_global_, Y_global_, and Z_global_ axes with the origin at the rear point of the home plate (Fig 3). The Y_global_ axis was directed from the home plate to the pitcher’s plate, and the Z_global_ axis indicated a vertically upward direction. The X_global_ axis was defined as the cross product of the Y_global_ and Z_global_ axes. For analysis of left-handed batters, a left-hand coordinate system with the same Y_global_ and Z_global_-axes as the right-hand coordinate system was utilized. To test the accuracy and reliability of this measurement method, one investigator digitized two reference markers on a swinging bat for five frames on two separate occasions. There were differences between the actual value (450 mm) and calculated value (mean ± standard deviation [*SD*] = 451 ± 0.3 mm). For the test-retest reliability of the distance, the coefficient of correlation was 0.973.

**Fig 4:**
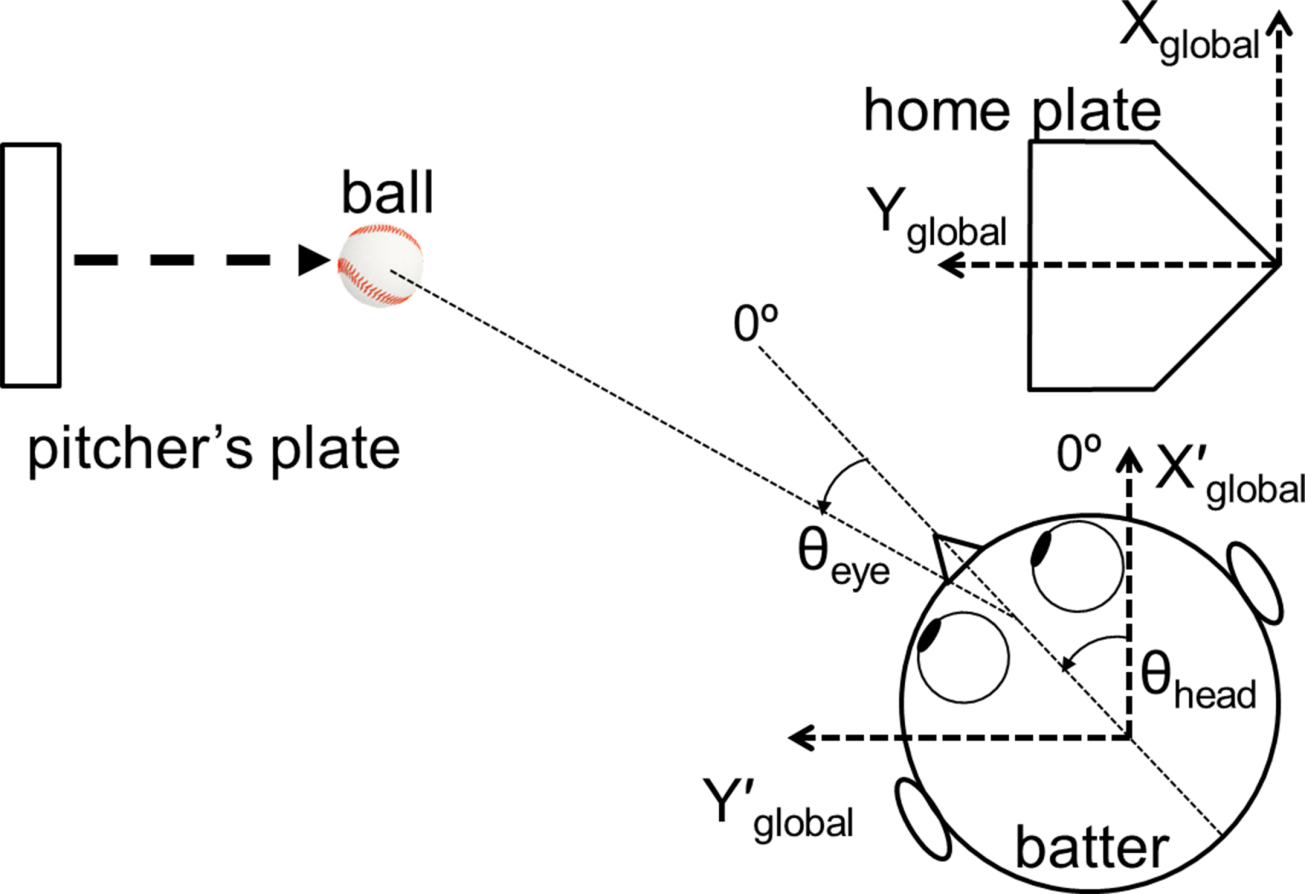
Eye (θ_eye)_ and head angles (θ_head_) on the global XY plane when the batters observed a moving baseball.

The horizontal movement of the batter’s head was calculated based on the orientation of the three markers, which were projected on the transverse plane. The head’s motion was filtered using a Butterworth filter with a cut-off frequency of 10 Hz. The EOG signal of each participant was standardized based on the EOG activity when they gazed at horizontal fixation points that were 60° lateral to the midline that was directly in front of them. When the participant faced the home plate and the direction of their head was parallel to the X_global_ axis, the head angle (θ_head_) was set at 0° (Fig 4). When the batter faced the pitching machine and the direction of their head was parallel to the Y_global_ axis, the two-dimensional angle of the head was defined as 90°. When the batter’s eyes looked straight forward, the eye angle (θ_eye_) was set at 0° (Fig 4). For right-handed batters, when the direction of their gaze moved right, the angle of the eyes decreased. For left-handed batters, the angle of the eyes decreased when their gaze moved left. The linear velocity of the bat was obtained from the average displacement of the bat end that were digitized from 5 to 1 ms before the moment of ball-bat contact.

### Statistical analysis

The average horizontal angular velocity of the eye and head movements during the four time periods (I-40 = 21–40% of total ball-flight, I-60 = 41–60% of total ball-flight, I-80 = 61–80% of total ball-flight, I-100 = 81–100% of total ball-flight) in each task was compared. These data were analysed using a three-way analysis of variance (ANOVA) with repeated measures using ball speed (fast vs. slow), body (eye vs. head), and time (the I-40, I-60, I-80, and I-100) as within-subjects factors. When significant effects were identified, a Tukey post-hoc multiple-comparison was adjusted to identify differences among the time periods. The statistical analyses were conducted with IBM SPSS statistical software, Windows version 20.0 (Japan IBM, Japan). Significance was set at an alpha level of *p* < 0.05.

## Results

**Fig 5:**
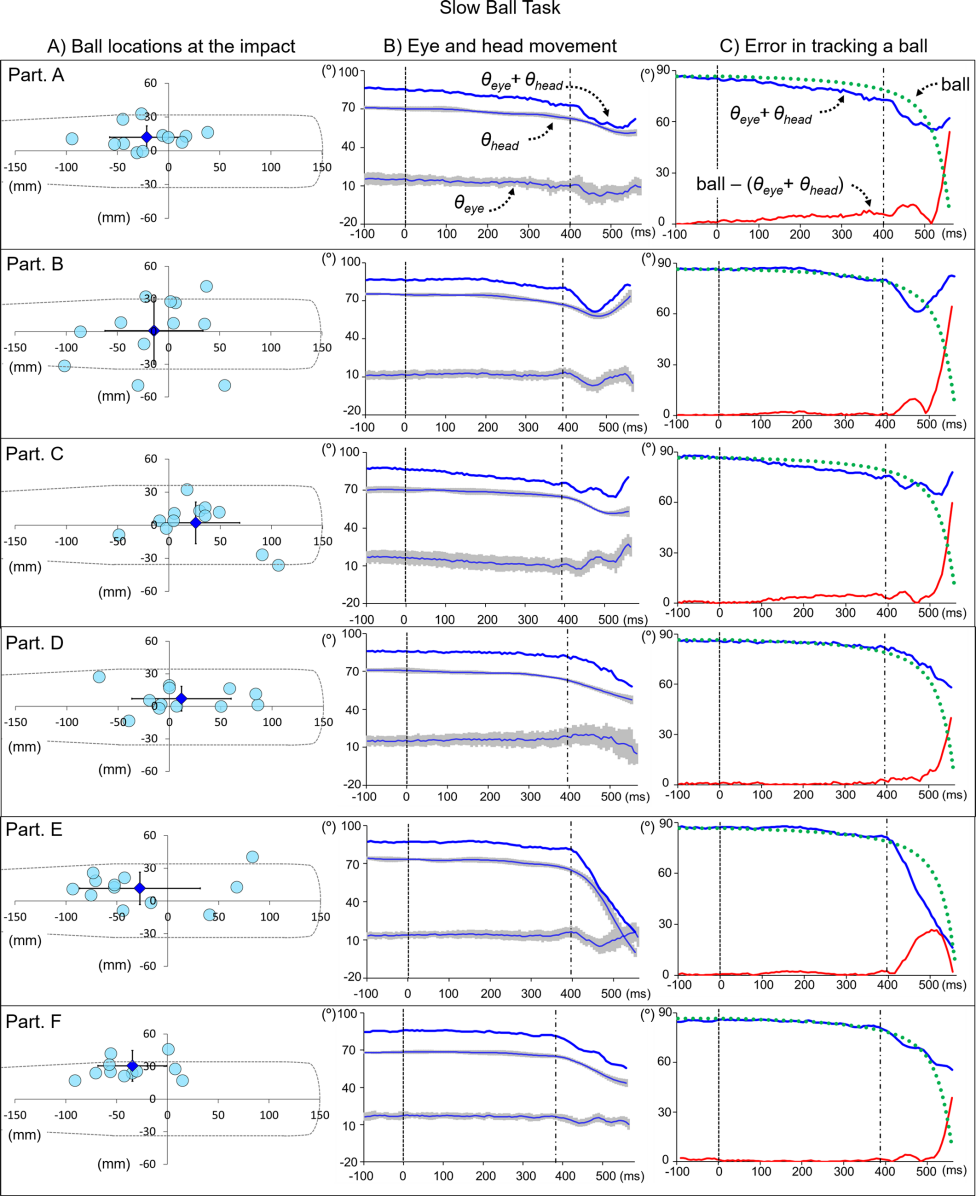
Ball-bat contact and directions of head, eye, and ball in Slow Ball Task. A) The left column indicates each participant’s ball-bat contact location, with the mean and standard deviation. B) The middle column indicates each participant’s θ_eye_, θ_head_ and resultant direction (θ_eye_+ θ_head_) from the ball’s release to its contact with the bat during the Slow Ball Task. C) The right column indicates the resultant direction, estimated direction of the launched ball (ball), and difference between the resultant direction and ball direction from its release to its contact with the bat. The dashed line at 0 ms indicates the time the ball was released, and the dash-dot line indicates the time at which the bat swing was expected to be initiated.

**Fig 6:**
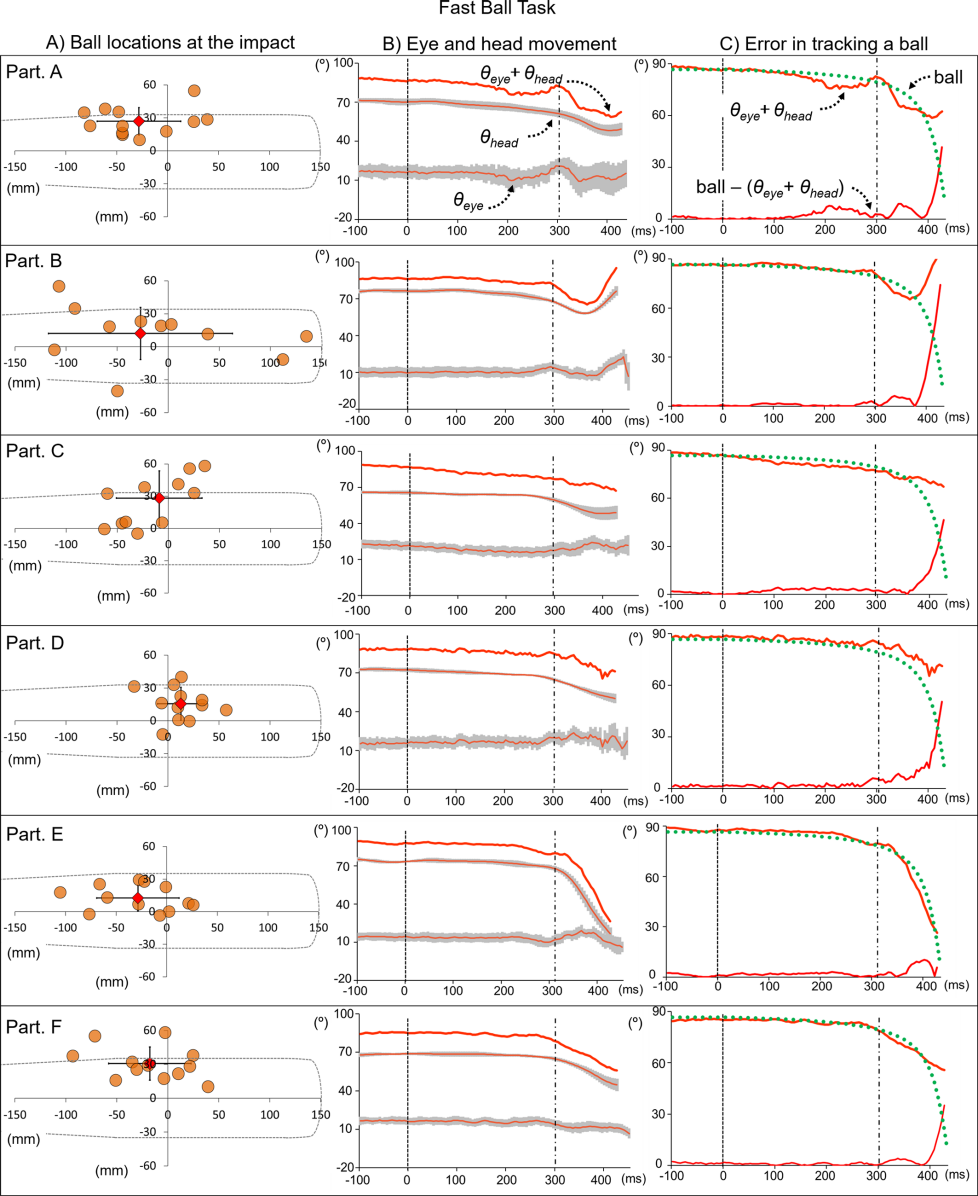
Ball-bat contact and directions of head, eye, and ball in Fast Ball Task. A) The left column indicates each participant’s ball-bat contact locations, with the mean and standard deviation. B) The middle column indicates each participant’s θ_eye_, θ_head_ and resultant direction (θ_eye_+ θ_head_) from the ball’s release to its contact with the bat during the Slow Ball Task. C) The right column indicates the resultant direction, estimated direction of the launched ball (ball), and difference between the resultant direction and ball direction from its release to its contact with the bat. The dashed line at 0 ms indicates the time at which the ball was released, and the dash-dot line indicates the time at which the bat swing was expected to be initiated.

The length of the time from the ball’s release to its contact with the bat was 543.0 ± 5.6 ms for the Slow Ball Task and 451.3 ± 3.6 ms for the Fast Ball Task (Table 1). The locations of the ball’s center at the moment of ball-bat contact in the Slow and Fast Ball Tasks are shown in Figs 5A and 6A, respectively. In both speed settings, all participants were able to make contact with the launched ball in all trials. The mean directions of the eye and head (θ_eye_ & θ_head_) for each participant’s task are shown in Figs 5B and 6B. Between the ball’s release and initiation of the bat swing, the angles of both the head and eyes were slightly and gradually decreased. Around the time of the bat swing, greater changes in the direction of the head and eyes, with individually distinctive patterns, were observed. The participant’s head and eyes moved in the same direction as that of the ball, but when they started their bat swing, differences in the patterns of head and eyes movements became more obvious. Differences between the ball’s estimated location from the batter’s perspective and the sum of the θ_eye_ and θ_head_ angles are shown in Figs 5C and 6C. The difference between the spatial eye direction (“θ_eye_ + θ_head_” in Figs 3C and 4C) and location of the ball from the batter’s view (“ball” in Figs 3C and 4C) was relatively small until the participant initiated the bat swing (“ball–[θ_eye_ + θ_head_]”). Participants A, B, and C stopped moving their heads while tracking the ball before it made contact with the bat, while participants D, E, and F kept turning their heads toward the tracking direction.

**Table 1:** Hitting performance of each participant regarding the ball-bat contact location, contact time, and bat head velocity.

		ball-sweet spot distance (mm)				ball release to contact (ms)		bat head velocity (m/s)		Bat swing time (ms)	
		horizontal		vertical							
	participant	distance	SD	distance	SD	time	SD	velocity	SD	time	SD
Slow Ball Task	A	-21.4	36.2	12.0	32.0	544	2.6	32.0	1.0	145	2.6
	B	-14.2	47.9	0.7	33.1	545	3.1	33.1	1.1	153	5.1
	C	26.2	42.7	2.1	35.9	548	2.9	35.9	1.3	155	2.5
	D	11.7	48.3	7.0	31.1	544	3.9	31.1	0.8	149	3.6
	E	-27.4	59.2	11.5	34.2	545	2.8	34.2	1.2	148	2.3
	F	-34.4	34.0	30.6	31.3	532	2.8	31.3	0.6	153	4.1
Fast Ball Task	A	-28.2	41.1	26.9	31.2	451	3.0	31.2	1.1	145	2.3
	B	-27.2	90.1	12.1	34.1	446	9.2	34.1	1.0	151	3.1
	C	-8.9	41.9	28.3	36.2	451	3.0	36.2	0.9	154	3.5
	D	12.6	22.9	15.5	31.1	450	2.0	31.1	1.3	147	3.3
	E	-28.5	40.7	12.6	34.8	457	3.6	34.8	0.9	148	4.3
	F	-17.6	40.5	30.7	30.8	453	2.6	30.8	1.1	152	4.2

SD: standard deviation

**Fig 7:**
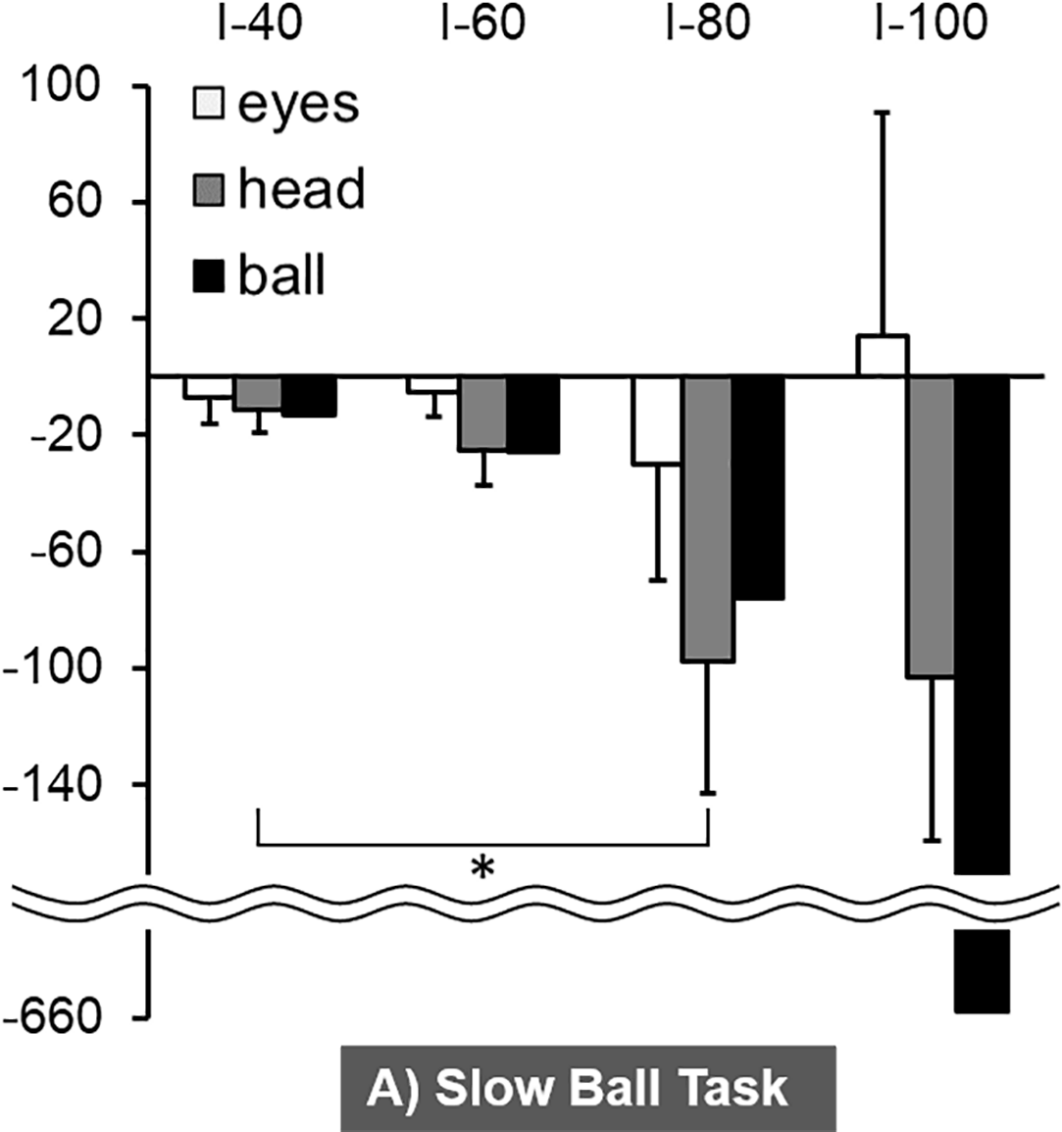
The mean horizontal angular velocity of the eyes and head during the four time periods of the Slow Ball Task. * *p* < 0.05.

**Fig 8:**
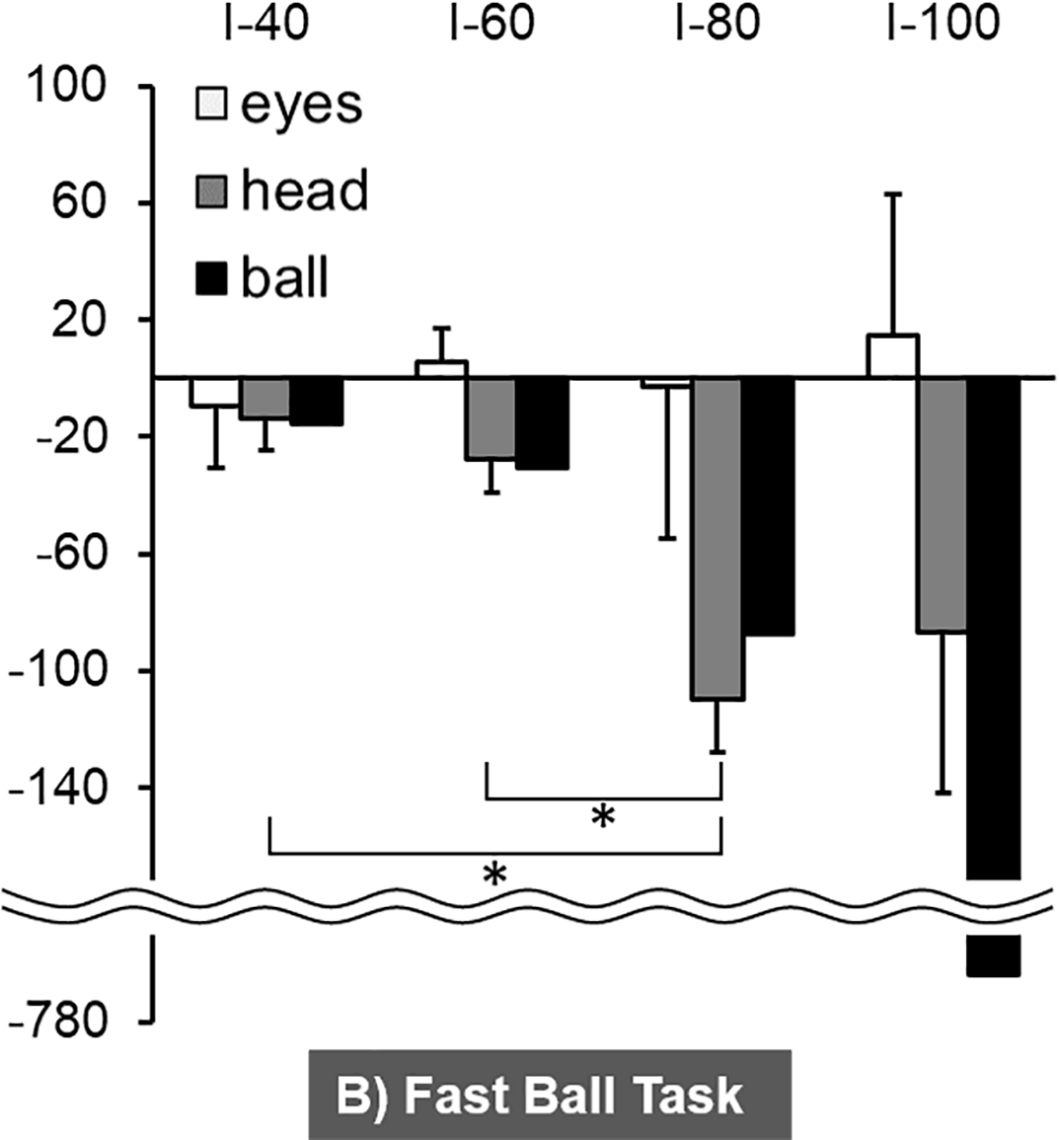
The mean horizontal angular velocity of the eyes and head during the four time periods of the Fast Ball Task. ** *p* < 0.01.

The three-way ANOVA results indicated significant main effect of body (F_1, 5_ = 76.4, *p* < 0.001) and significant interaction between body and time period (F_3, 15_ = 4.9, *p* < 0.05), while there was no significant effect of ball speed. The mean horizontal angular velocity of the eyes and head during the Slow Ball Task are shown in Fig 7 and S1 Table. The Tukey post-hoc multiple-comparison results showed that the horizontal angular velocity of the head during I-80 was significantly faster than that during I-40 (*p* < 0.05). In the Fast Ball Task, the Tukey post-hoc multiple-comparison test showed that the horizontal angular velocity of the head during I-80 was significantly faster than that during I-40 and I-60 (*p* < 0.05) (Fig 8).

## Discussion

In this study, we examined the head-eye movements of experienced baseball batters while they hit a launched fastball. We found that while the tracking motion of the head became faster as the launched ball came close to the batters and they started to swing the bat, there was no change in the tracking angular motion of the eyes. In this study, the batter’s use of both head and non-saccadic eye movements during hitting supports the results of a previous report by Bahill and LaRitz [8]. From the batter’s perspective, although the rate of horizontal angular displacement of a launched ball increases as it moves closer to the batter, a significant increase in the horizontal angular velocity of the head was found only during the I-80 period in both the Slow Ball Task and Fast Ball Task. In contrast, no significant differences in the angular velocity of the eyes were observed during the four time periods. These findings indicate that the eyes’ position relative to the head did not change as fast as the head’s direction did. Therefore, our results suggest that batters track a flying ball with head movements and without substantial eye movement. That is, conventional vision training with a wide range of saccadic or smooth-pursuit eye movements may not reflect the characteristics of tracking strategies that are used in baseball hitting.

### Head and eye movement before the bat swing was initiated

Visual information from ball release to 150 ms before the ball-bat contact, which is the moment for the bat’s acceleration [17], is crucial for accurate hitting. Based on the calculated displacement of a launched ball in the two tasks, horizontal angular displacement of the ball from the release to 150 ms before the ball-bat contact is less than 20°. Angular velocity of the ball at that point was approximately 78°·s^-1^ for the Slow Ball Task and approximately 66°·s^-1^ for the Fast Ball Task. The angular velocities that were determined during the Slow Ball and Fast Ball Tasks in this study approximately reached the physiological limit of the human eye [18]. However, the participants were able to track the ball by using not only eye movement but also head movement. Since there was no significant improvement in hitting accuracy when this factor was compared during the trials with visual information for the last 150 ms before the ball made contact with the bat and those without [7], the batters were able to track the ball and obtain necessary information for accurate hitting with steady coordinated movements of the head and eyes.

### Head and eye movement after the bat swing was initiated

Changes in the head and eye direction became larger at 61–80% of ball flight time. A more rapid increase in the ball’s angular velocity and/or bat swing motion may have evoked rapid change in the head and eye angles. Interestingly, the direction of the head’s angular movement at the beginning of the bat swing was opposite to the direction of the angular movement of the trunk. Head movements that are opposite to the rotation of the trunk may give batters additional time to see the ball and may counter-balance the angular momentum of the trunk’s rotation. In cricket, the superior abilities to couple the direction of the head to the moving ball and the predictive saccadic eye movement to the ball bounce and ball-bat contact location were reported as characteristics of elite cricket batters [11, 12]. However, in this study, individual differences in the angular movement of the head and eyes were more prominent when the batters swung the bat. For example, participants A, B, and C seemed to have stopped their heads’ motions while tracking the ball before it made contact with the bat, while participants D, E, and F kept turning their heads toward the tracking direction. In particular, participant E was able to align his gaze with the ball at the moment of ball-bat contact. However, neither of the tracking strategies was advantageous over the other based on the batters’ hitting performances. Therefore, batters might have different strategies for performing head and eye movements while they swing a bat. Sarpeshkar et al. revealed that the ability to align the gaze with the ball at contact was a predictor of straight-ball batting skill in cricket [19]. In the present study, only fastball was launched without mixing any balls outside of the strike zone, therefore, the task might not be difficult enough to discriminate the participants’ skill level.

For a successful interceptive action, humans adjust their motions based on their eye-centered reference frame [20]. In baseball hitting, the ball’s initial location is determined with the batter’s eye-centered coordinates, which are obtained using visual information. Based on the location of a thrown ball in the eye-centered coordinates, the spatial relationship between the ball and batter is determined with head-, body-, and joint-centered coordinates. However, if the batter’s eye position changes, the spatial relationship between the ball and batter in all of the coordinate systems must be updated based on the ball’s position on the latest retinal image. The greater the change in the eyes’ position is, the more the other coordinates must change. Thus, excessive changes in the eyes’ position may increase the batter’s chance of mis-hitting the ball because of a large error that is induced by either a shortage of time or excessive processing that is needed to determine the new spatial relationship between the ball and batter’s coordinate systems. Because of the large angular displacement of a thrown ball on the retinal image and the forceful rotational movement that is required to swing a bat, maintaining the image of the ball on a discrete locus in the retina might be impossible just before the ball makes contact with the bat. The vestibulo-ocular reflex is another complication. This reflex opposes the movement of the head and eyes in the same direction. To move the eyes and head simultaneously, the batter must suppress the vestibulo-ocular reflex. It is also possible that batters do not rely on visual information for the final 150 ms of the ball’s flight because rapid angular displacement of the ball requires a substantial change in the positions of the head and eyes to successfully track the ball. Steady and minimal eye movement might be a key requirement for batters to obtain optimal visual information and thus maximize the possibility that they will correctly estimate the ball’s future location. However, considering several factors, such as the limited response time, the complex process of coordinate transformation, and the existence of the vestibulo-ocular reflex, the execution of an ideal tracking action with little or no voluntary control must be acquired through extensive batting practice.

### Limitations of the study

In this study, the batters were instructed to hit balls that were launched from a pitching machine at consistent speeds. Their ball tracking strategy and swing motion might have differed from those in an actual hitting situation, which requires batters to distinguish various types and speeds of pitches. Another limitation was the lack of data for the vertical component of the head and eye movements. In future studies, it will be necessary to investigate two-dimensional tracking motions of the head and eyes when the batters interact with a real pitcher.

## Conclusion

Head-eye movement of experienced baseball batters while they are hitting launched fastball were examined. While tracking motion of the head became faster as launched ball came close to batters and they started the bat swing, there was no change in tracking angular motion of the eyes. This study suggests that rapid eye movement would not be used during fastball hitting to accurately estimate the ball’s future movements, based on the eye-centered coordinates.

## Supporting information

S1 Table The horizontal angular velocity of the eyes and head. Numerical values for the mean and standard deviations of horizontal angular velocity of the eyes and head during the four time periods.
(XLSX)
